# Supplementary material for: Performance Restoration of Chemically Recycled Carbon Fibres Through Surface Modification with Sizing
Source: Polymers (Basel). 2024 Dec 26;17(1):33. doi: 10.3390/polym17010033 (PMC11723044; doi:10.3390/polym17010033)
Supplement: Supplementary file 1 [file polymers-17-00033-s001.zip › polymers-3367817-supplementary.pdf]

1. SEM Support data

Ref_CF

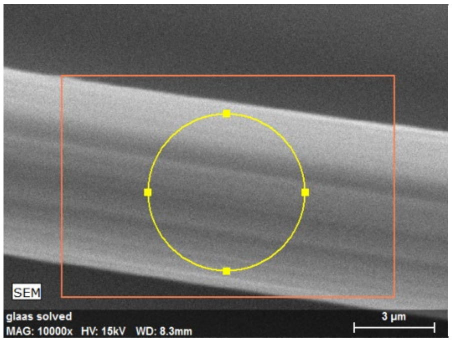

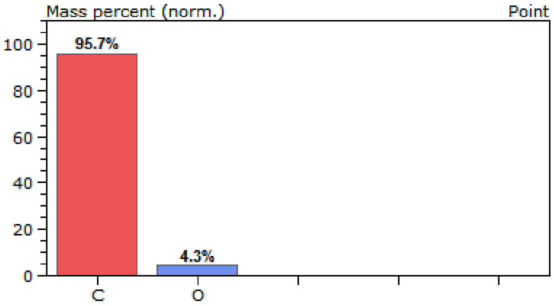

Ch_rCF

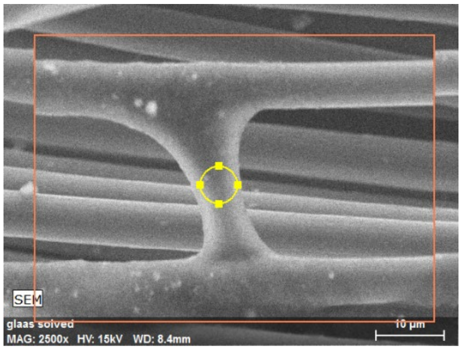

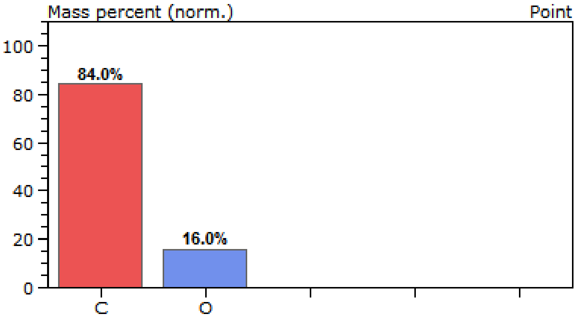

Pl_rCF

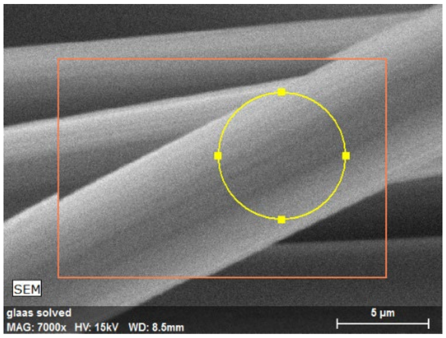

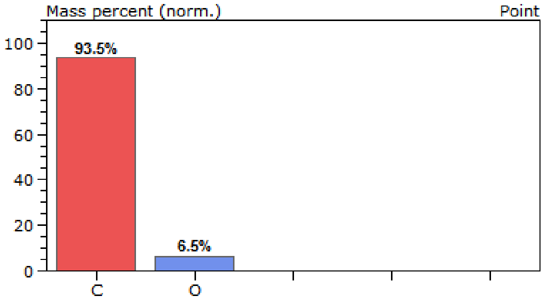

Sized_Ch_rCF

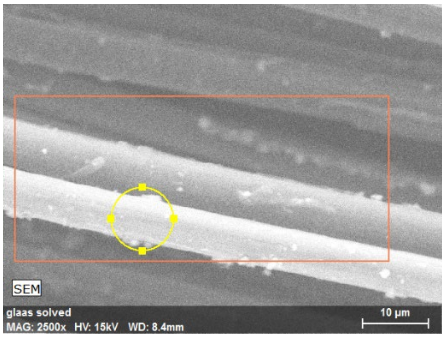

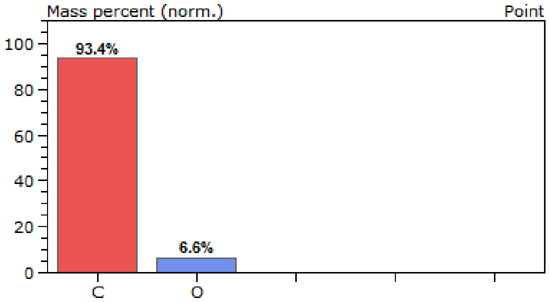

Sized_Pl_rCF

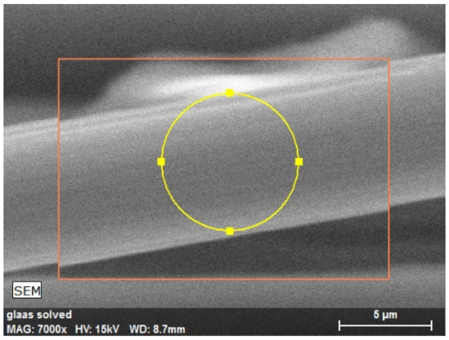

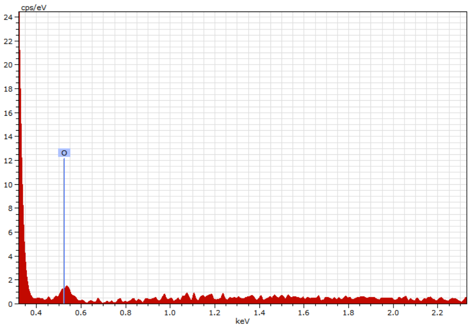

Raw data for extracting the percentages of each element are available.
